# 271. Determining Risk Factors Associated with an Increased Incidence of Pulmonary Embolism: A Multi-Center Study of 3,675 Patients

**DOI:** 10.1093/ofid/ofac492.349

**Published:** 2022-12-15

**Authors:** Lucas Wang, Mujahed Abualfoul, Kristopher Aten, Ariella Azimi, Victor Canela, Lawrence Hoang, Sri Prathivada, Michael Vu, Sherry Zhao, Manavjot Sidhu

**Affiliations:** Methodist Dallas Medical Center, Arlington, Texas; Methodist Dallas Medical Center, Arlington, Texas; Methodist Dallas Medical Center, Arlington, Texas; Methodist Dallas Medical Center, Arlington, Texas; Methodist Dallas Medical Center, Arlington, Texas; Methodist Dallas Medical Center, Arlington, Texas; Methodist Dallas Medical Center, Arlington, Texas; Methodist Dallas Medical Center, Arlington, Texas; Methodist Dallas Medical Center, Arlington, Texas; Methodist Dallas Medical Center, Arlington, Texas

## Abstract

**Background:**

Coronavirus Disease 2019 (COVID-19) is associated with an increased incidence of pulmonary embolism (PE). Both conditions increase hospital complications and mortality, especially when exhibited concurrently. Unfortunately, both conditions may present similarly, and physicians often have a difficult time finding clinical indicators to suggest pursuing further evaluation of a PE during a COVID-19 infection.

**Methods:**

Using a multi-center facility database, we conducted a retrospective analysis of 3,675 COVID-19 patients at Methodist Health System from March 2020 to December 2020. COVID-19 infection was determined via molecular PCR testing and PE was determined by computed tomography (CT) scan with angiography. Patient demographics and laboratory values were determined by a manual review of patient charts. Chi-Square test was used to analyze observed variables. Odds ratios were calculated for variables with a statistically significant difference (p < 0.05).

**Results:**

Of the 3,675 patients diagnosed with COVID-19, 150 (4.1%) were diagnosed with PE. Elevated D-dimer level had a statistically significant association with increased rate of PE (OR 0.1988, 95% CI 0.0727 – 0.5438, p < 0.001). Factors such as elevated C-reactive protein (p = 0.61), IL-6 (p = 0.26), smoking history (p = 0.70), age over 65 (p=0.54), BMI over 25 (p = 0.42), and history of chronic kidney disease (p = 0.16) did not show a significant association with PE incidence. Of note, patients with PE during admission were seen to have an increased incidence of intubation (OR 0.40, 95% CI 0.2660 – 0.6029, p < 0.001).

**Conclusion:**

Our study suggests that COVID-19 patients with elevated D-dimer have higher odds of having a PE. This study also suggests that COVID-19 patients that develop a PE during hospitalization are more likely to require intubation.

**Disclosures:**

**All Authors**: No reported disclosures.

